# Supplementary material for: Genomic diversity and ecology of human-associated Akkermansia species in the gut microbiome revealed by extensive metagenomic assembly
Source: Genome Biol. 2021 Jul 14;22:209. doi: 10.1186/s13059-021-02427-7 (PMC8278651; doi:10.1186/s13059-021-02427-7)
Supplement: Supplementary file 2 — Additional file 2. Supplementary Figures S1 to S11. [file 13059_2021_2427_MOESM2_ESM.pdf]

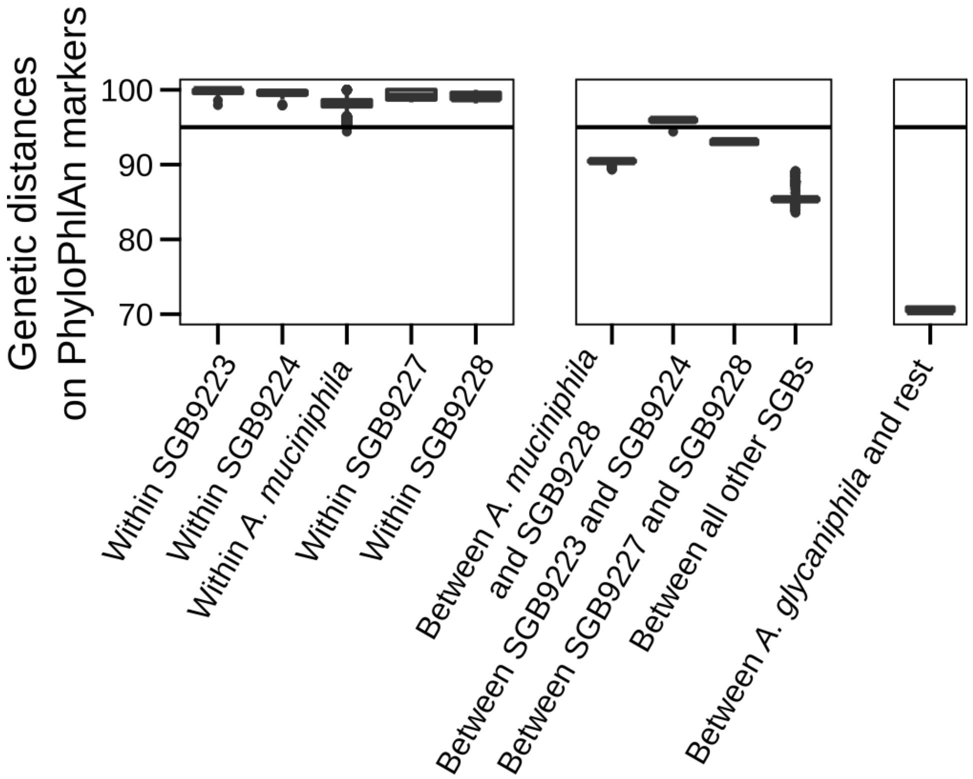

Fig. S1: Within- and between-clade whole-genome genetic distance estimates obtained using PhyloPhlAn 3 [47]. Related to **Fig. 1**.

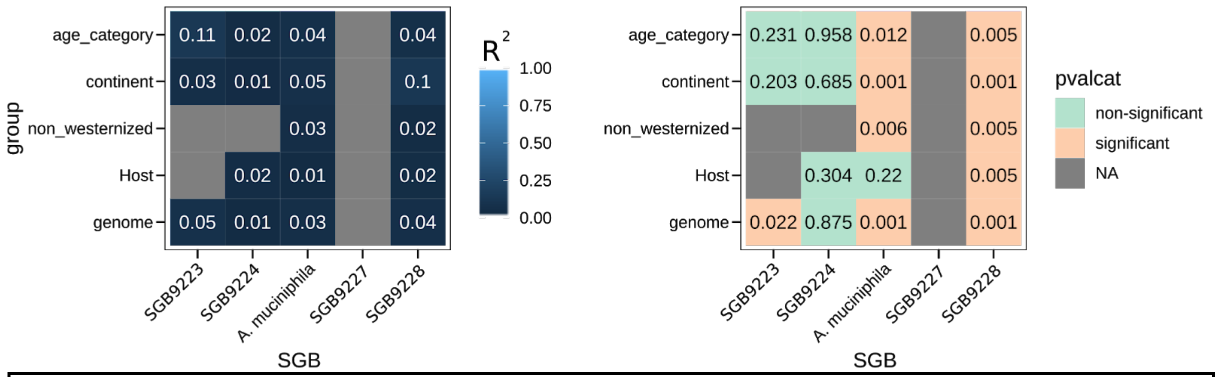

Fig. S2: For each SGB and each considered metadata, we performed a PERMANOVA test on pairwise distances. PERMANOVA tests were performed using the Adonis function from the vegan R package. SGB9227 was omitted because of its small size.

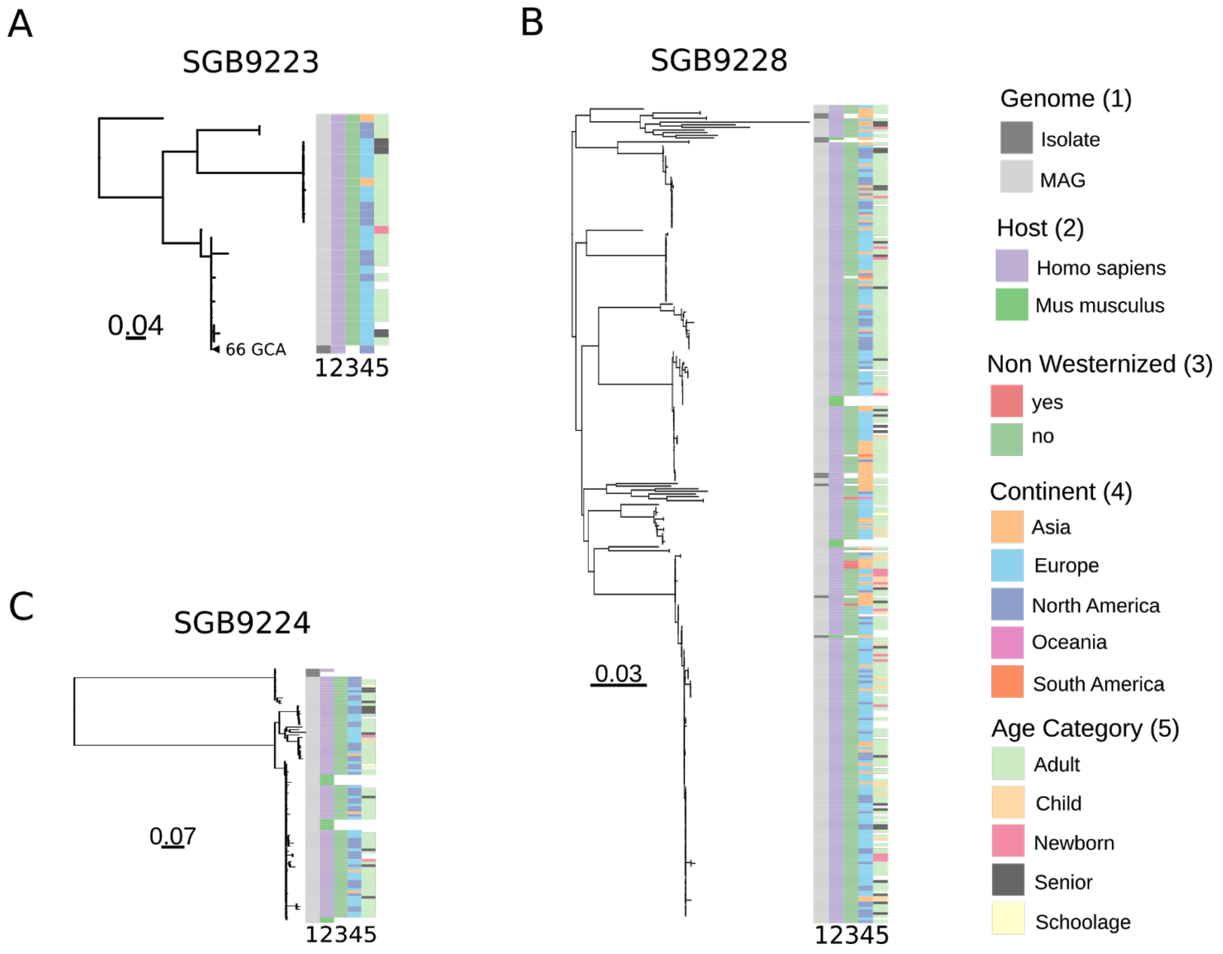

Fig. S3: Phylogenetic trees of *Akkermansia* candidate species built using core genes (see **Methods**). SG9227 is not shown due to its small size. The core genes used for each tree are in at least 80% of the genomes in candidate species, respectively 1131, 799 and 996 for SGB9223, SGB9224, and SGB9228.

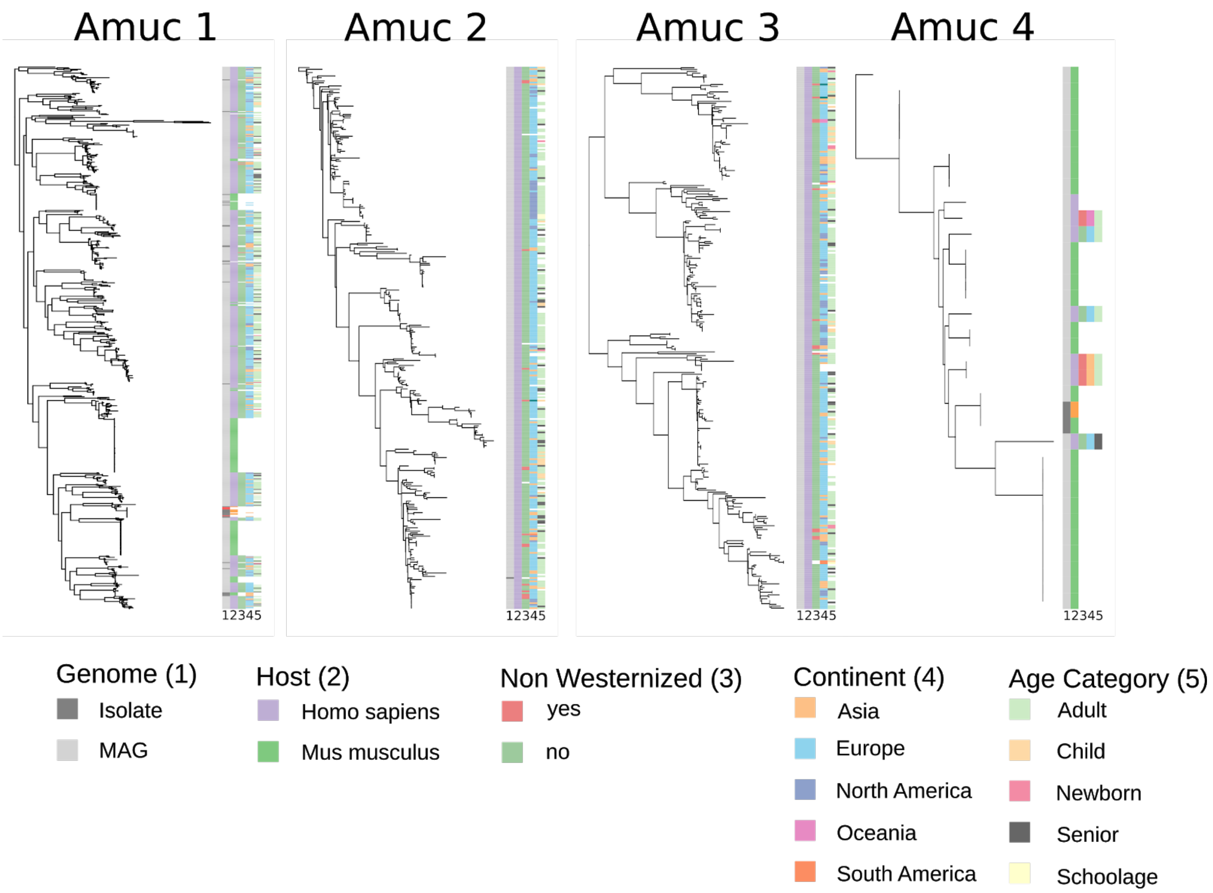

Fig. **S4**: Phylogenetic tree of *A. muciniphila* subspecies built using 169 core genes (see **Fig. 4, Methods**).

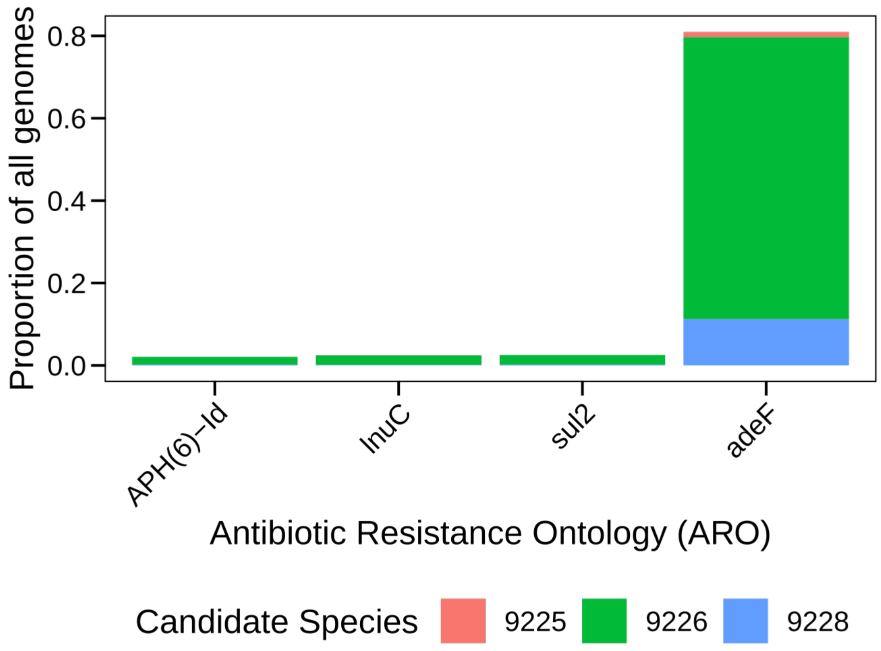

Fig. S5: We annotated all genomes using the resistance gene identifier (RGI) software (https://github.com/arpcard/rgi). Hits above the bit score threshold that covered at least 70% of the reference sequence in CARD were kept. Only hits in the ARO that are found in at least 1% of genomes are shown.

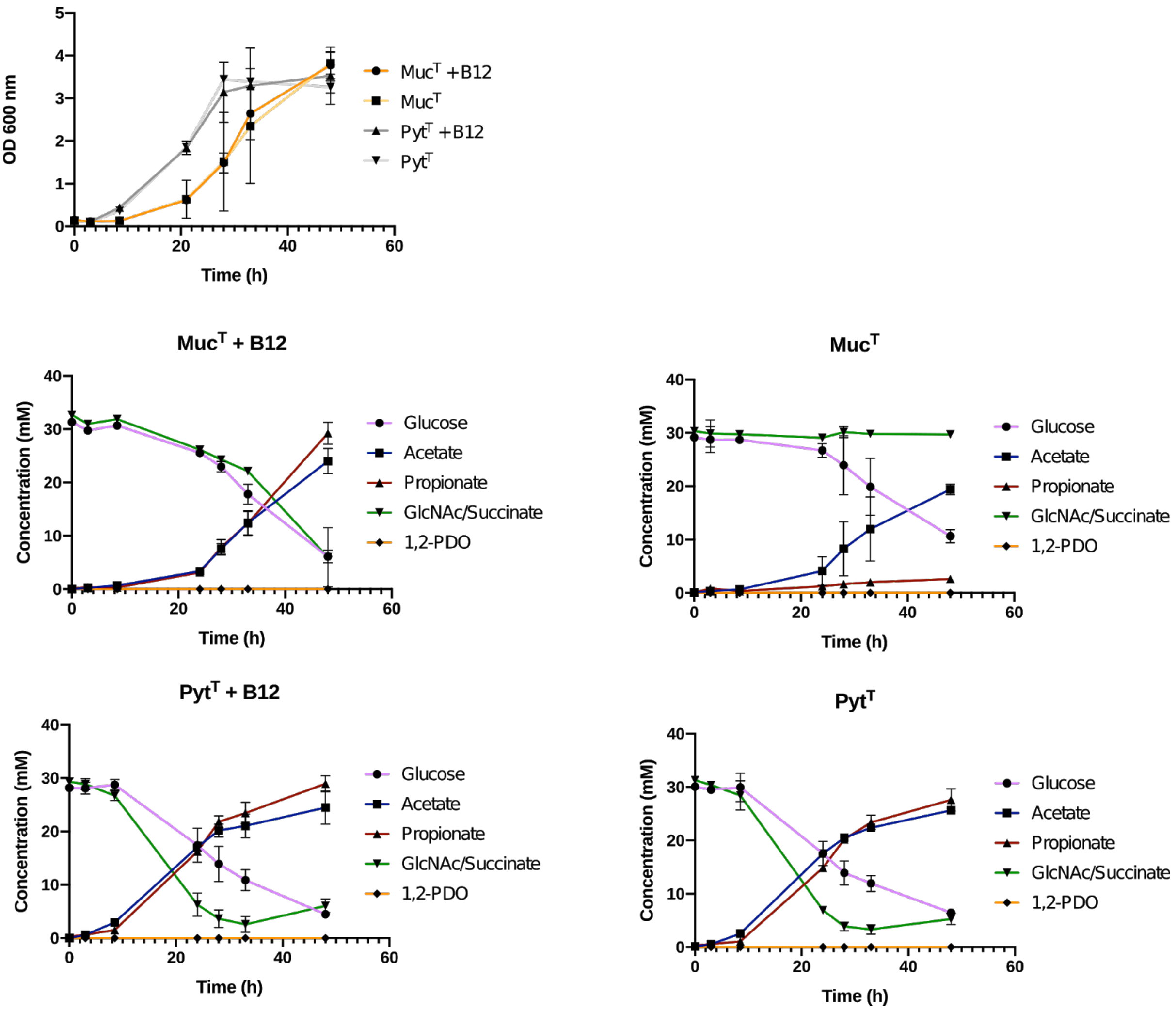

Fig. S6: (A) Growth curve of *A. muciniphila* Muc^T and *A. glycaniphila* Pyt^T in minimal medium in the presence and absence of vitamin B12. Metabolites produced and substrates utilized by both Muc^T (B) and Pyt^T(C) both in the presence and absence of vitamin B12 were measured using HPLC, with propionate being a proxy for B12 production and utilization as its production is dependent on the B12-dependent methyl-malonyl CoA synthase reaction [57].

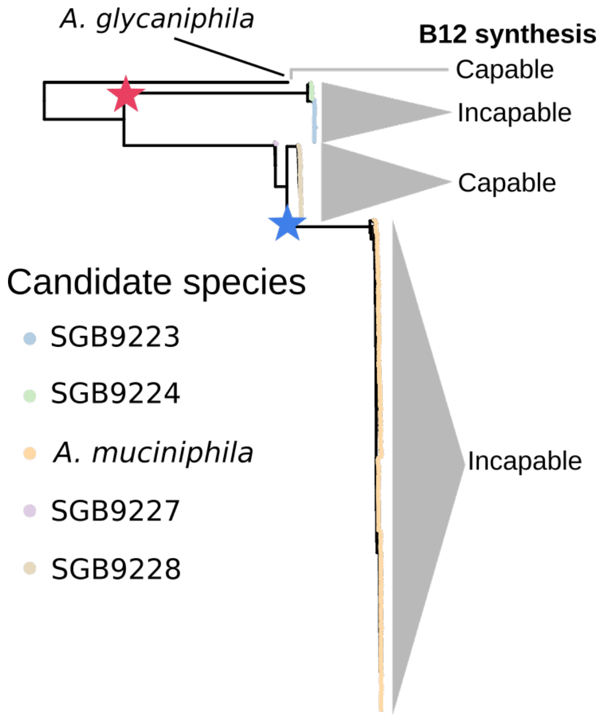

Fig. S7: Phylogenetic tree of all candidate species (including *A. glycaniphila*) annotated with B12 synthesis capabilities. Stars indicate two putative loss of vitamin B12 biosynthesis capability events in the most recent common ancestor of SGB9223/SGB9224 (red star) and *A. muciniphila* (blue star).

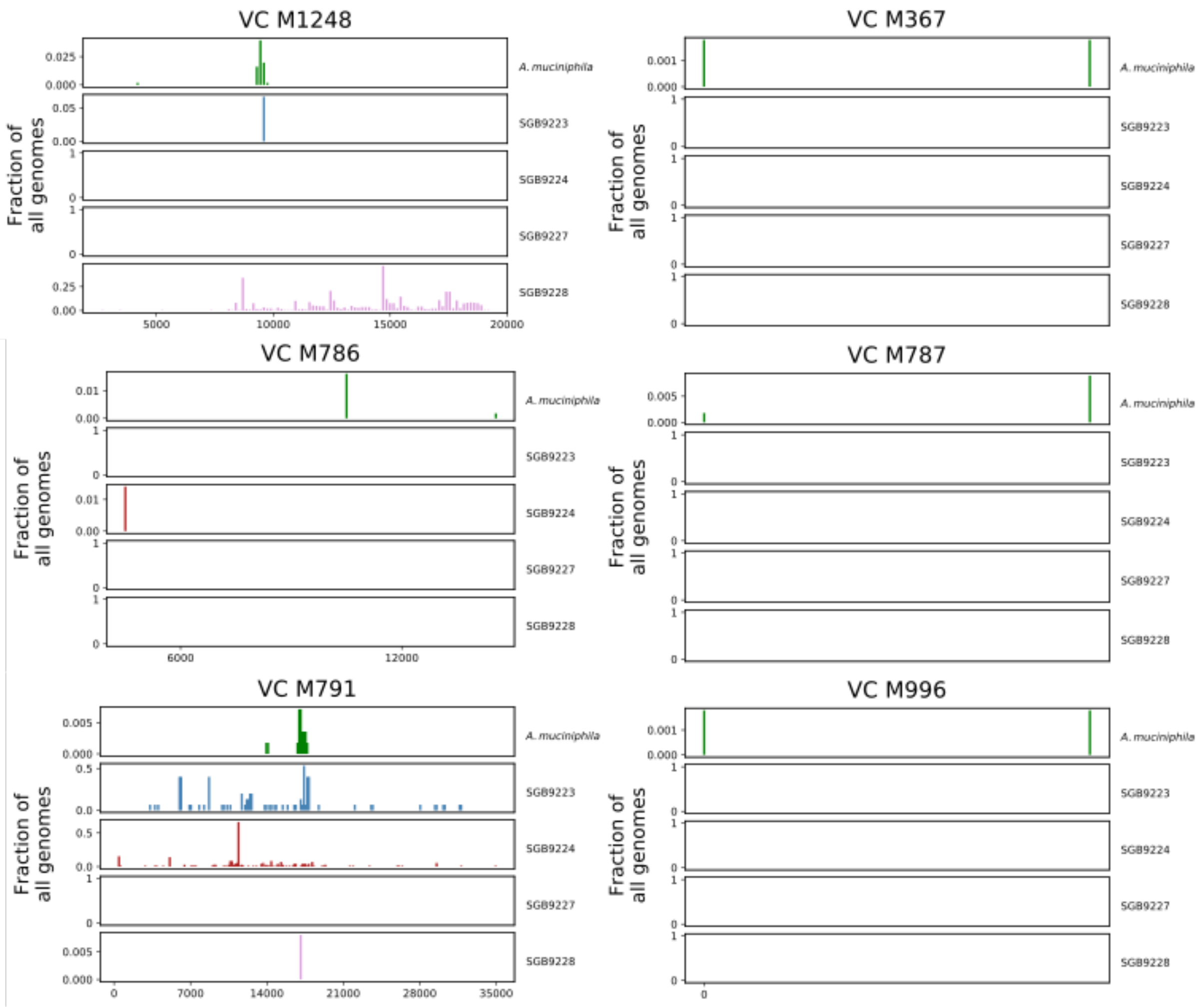

Fig. S8: Maps of spacers from *Akkermansia* genomes against six VCs, visualized with a sliding window of 150 nt. See **Fig. 3G**.

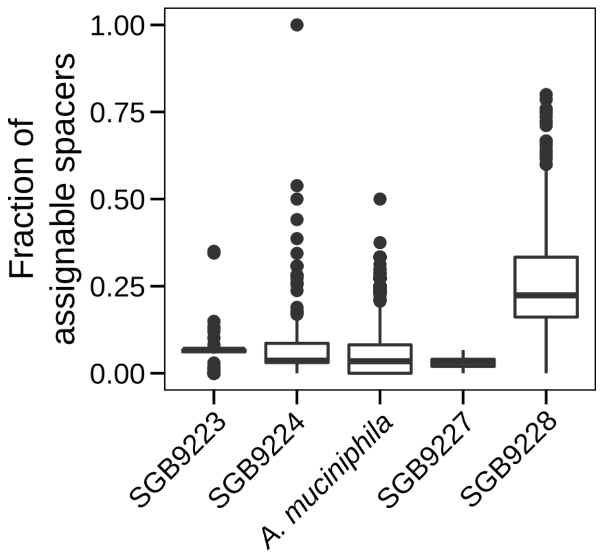

Fig. S9: The total fraction of assignable spacer sequences (those with a near-perfect match against a gut phage, see **Methods**) per *Akkermansia* candidate species.

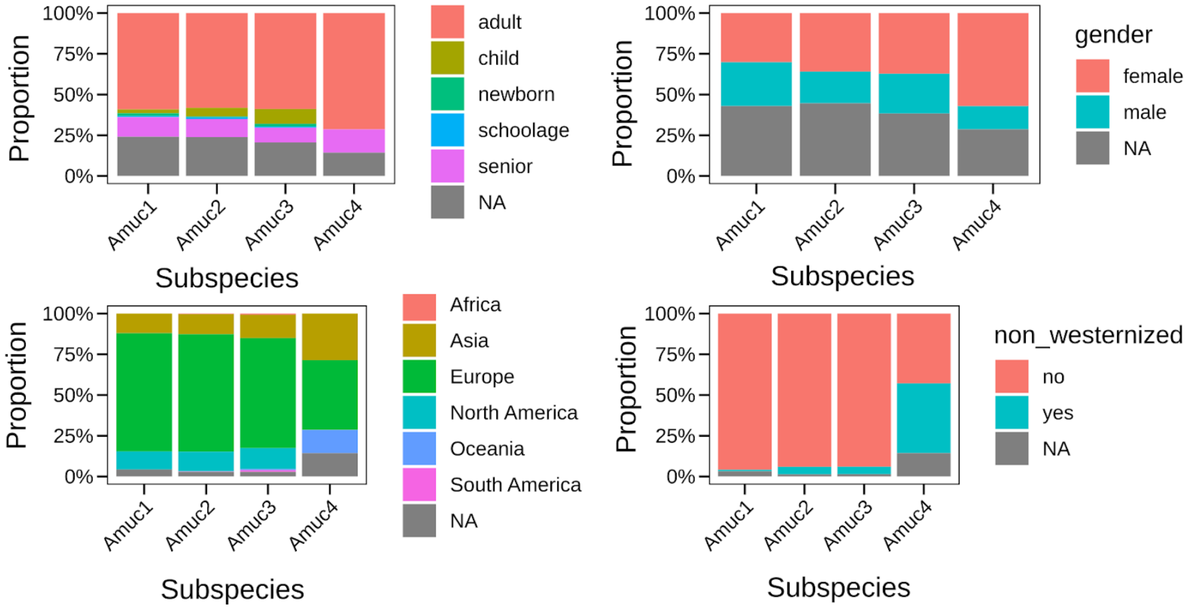

Fig. S10: Barplots showing the distribution of *A. muciniphila* subspecies for host age, gender, continent (origin) and Westernization status.

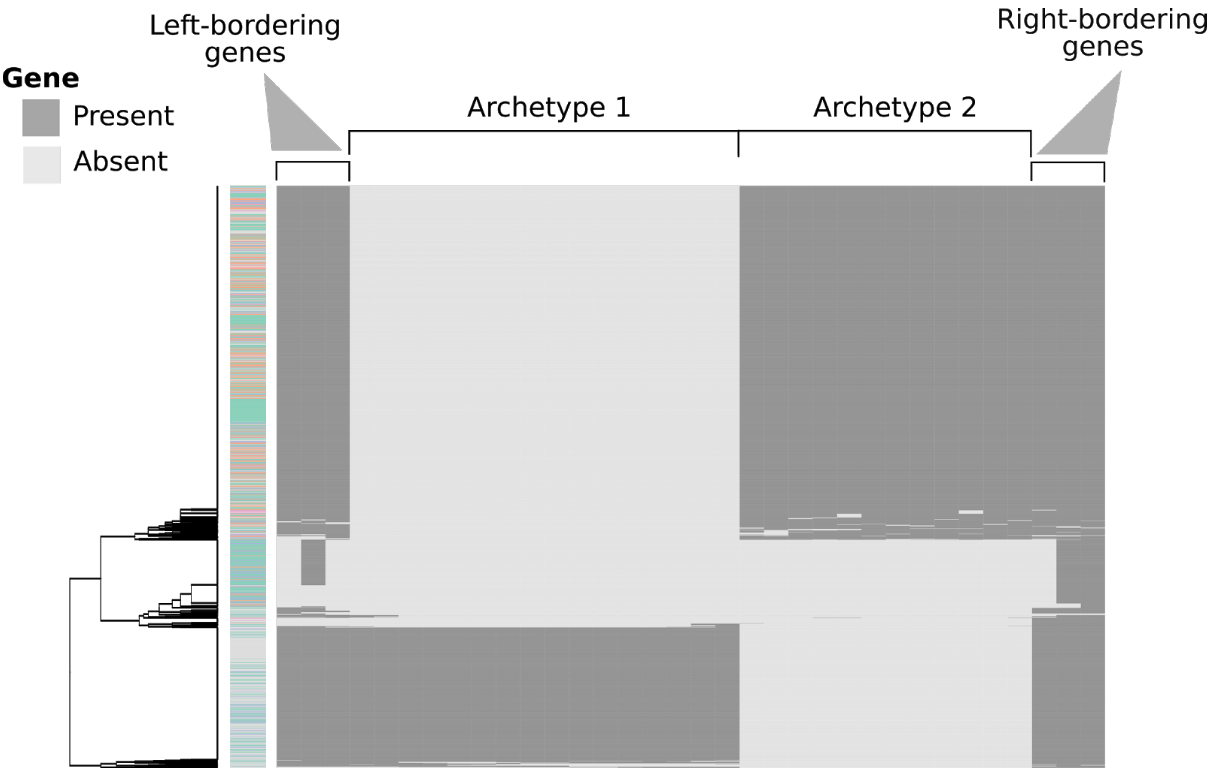

Fig. S11: Gene presence/absence heatmap for both operon archetypes as well as three left- and right-bordering genes. Most MAGs have either one or the other archetype as well as both left- and right-bordering genes, whereas a small fraction of MAGs has neither operon and also only some bordering genes. Related to **Fig. 5**.
